# Supplementary material for: Shorter birth intervals between siblings are associated with increased risk of parental divorce
Source: PLoS One. 2020 Jan 31;15(1):e0228237. doi: 10.1371/journal.pone.0228237 (PMC6993964; doi:10.1371/journal.pone.0228237)
Supplement: S1 Table — (PDF) [file pone.0228237.s001.pdf]

S1 Table. Hazard ratios from Cox regressions predicting parental risk of divorce by all the covariates (mutually adjusted) in individuals with two and three children.

	2 children		3 children	
	HR	95 % CI	HR	95 % CI
Birth cohort				
1955 - 59 (ref.)	1.00	-	1.00	-
1960 - 64	1.10	1.03, 1.17	1.05[a]	.95, 1.15
1965 - 69	1.38	1.29, 1.47	1.19[b]	1.06, 1.32
1970 -74	1.58	1.46, 1.71	1.41	1.22, 1.62
1975 - 79	1.35	1.17, 1.56	1.59	1.21, 2.10
Sex (ref. = women)	1.29	1.23, 1.36	1.29	1.19, 1.41
AFR	.91	.91, .92	.91	.90, .92
Marriage length (years)	.80	.78, .83	.82	.78, .86
Marriage length (quadratic)	1.01	1.01, 1.01	1.01	1.01, 1.01
Timing of marriage				
Married before 1st child (ref.)	1.00		1.00	
Married before 2nd child	1.06[a]	1.00, 1.12	1.20	1.08, 1.33
Married before 3rd child			1.06[a]	.91, 1.24
SES at age 35				
Farmer	.47	.38, .58	.50	.39, .65
Entrepreneur	1.12[a]	1.00, 1.25	1.03[a]	.86, 1.24
Upper white collar (ref.)	1.00	-	1.00	-
Lower white collar	1.09[b]	1.01, 1.17	1.09[a]	.95, 1.24
Manual worker	1.16	1.08, 1.26	1.19[b]	1.04, 1.36
Student	1.61	1.40, 1.85	1.71	1.37, 2.13
Unknown	1.87	1.70, 2.05	1.68	1.44, 1.96
Missing	1.93	1.67, 2.24	1.76	1.35, 2.31

Note. All p-values <.001, except [a]p >.05 (not significant); [b]p<.05.
CI = Confidence interval; AFR = Age at first reproduction; SES = Socioeconomic status.

Supporting Table S1 for Berg V. et al.: Shorter birth intervals between siblings are associated with increased risk of parental divorce; PlosOne 2020
