# Supplementary material for: Shorter birth intervals between siblings are associated with increased risk of parental divorce
Source: PLoS One. 2020 Jan 31;15(1):e0228237. doi: 10.1371/journal.pone.0228237 (PMC6993964; doi:10.1371/journal.pone.0228237)
Supplement: S2 Table — (PDF) [file pone.0228237.s002.pdf]

Table S2. Results from Cox regressions predicting the risk of divorce by interbirth interval (IBI) between children in two-child families, with varying reference categories.

	Model 1		Model 2		Model 3		Model 4		Model 5	
	HR	p	HR	p	HR	p	HR	p	HR	p
IBI										
< 18 months	1.00 (ref)		1.26	<.001	1.44	<.001	1.65	<.001	1.49	<.001
18-24	.79	<.001	1.00 (ref.)		1.15	<.001	1.31	<.001	1.18	.001
24-30	.69	<.001	.87	<.001	1 (ref.)		1.15	.002	1.03	.549
30-36	.60	<.001	.76	<.001	.87	.002	1.00 (ref)		.90	.028
36-42	.67	<.001	.85	.001	.97	.549	1.11	.028	1.00 (ref)	
42-48	.62	<.001	.78	<.001	.90	.053	1.03	.617	.92	.171
48-54	.57	<.001	.72	<.001	.83	.003	.95	.407	.85	.014
54-60	.65	<.001	.82	.008	.94	.420	1.08	.266	.97	.684
60-66	.66	<.001	.83	.028	.95	.553	1.09	.285	.98	.803
66-72	.67	<.001	.84	.083	.96	.698	1.10	.322	.99	.919
> 72	.63	<.001	.80	.003	.91	.214	1.05	.521	.94	.377

Note. All models control for birth cohort, marriage length at the start of follow-up, sex, age at first reproduction, and timing of marriage.

Supporting Table S2 for Berg V. et al.: Shorter birth intervals between siblings are associated with increased risk of parental divorce; PlosOne 2020
