# Supplementary material for: Shorter birth intervals between siblings are associated with increased risk of parental divorce
Source: PLoS One. 2020 Jan 31;15(1):e0228237. doi: 10.1371/journal.pone.0228237 (PMC6993964; doi:10.1371/journal.pone.0228237)
Supplement: S3 Table — (PDF) [file pone.0228237.s003.pdf]

S3 Table. Hazard ratios (HR) from Cox regression predicting risk of divorce in individuals with three children by first and second birth interval and their interaction.

	HR (95% CI)	p
1st IBI		
≤ 18 months	Ref.	
18.01 - 24	0.95 (0.57, 1.59)	.844
24.01 - 36	0.59 (0.36, 0.97)	.039
36.01 - 48	0.78 (0.44, 1.38)	.397
> 48	0.82 (0.48, 1.40)	.461
2nd IBI		
≤ 18 months	Ref.	
18.01 - 24	1.37 (0.86, 2.18)	.183
24.01 - 36	1.09 (0.70, 1.68)	.709
36.01 - 48	0.83 (0.52, 1.33)	.436
> 48	0.90 (0.57, 1.43)	.663
Interaction between 1st IBI of 18.01-24 months and 2nd IBI of		
18.01 - 24	0.63 (0.34, 1.17)	.141
24.01 - 36	0.74 (0.42, 1.31)	.309
36.01 - 48	0.90 (0.49, 1.63)	.719
> 48	0.92 (0.53, 1.59)	.752
Interaction between 1st IBI of 24.01-36 months and 2nd IBI of		
18.01 - 24	0.90 (0.49, 1.63)	.722
24.01 - 36	1.02 (0.58, 1.78)	.945
36.01 - 48	1.45 (0.81, 2.58)	.211
> 48	1.36 (0.79, 2.32)	.263
Interaction between 1st IBI of 36.01-48 months and 2nd IBI of		
18.01 - 24	0.86 (0.45, 1.67)	.662
24.01 - 36	1.04 (0.56, 1.95)	.894
36.01 - 48	1.33 (0.70, 2.53)	.392
> 48	0.98 (0.54, 1.80)	.957
Interaction between 1st IBI of > 48 months and 2nd IBI of		
18.01 - 24	0.65 (0.35, 1.21)	.175
24.01 - 36	1.21 (0.69, 2.15)	.508
36.01 - 48	0.97 (0.51, 1.84)	.934
> 48	0.95 (0.53, 1.69)	.855

Note. The model additionally controls for birth cohort, marriage length at the birth of third child, sex, age at first reproduction and timing of marriage.

Supporting Table S3 for Berg V. et al.: Shorter birth intervals between siblings are associated with increased risk of parental divorce; PlosOne 2020
