# Supplementary material for: Shorter birth intervals between siblings are associated with increased risk of parental divorce
Source: PLoS One. 2020 Jan 31;15(1):e0228237. doi: 10.1371/journal.pone.0228237 (PMC6993964; doi:10.1371/journal.pone.0228237)
Supplement: S4 Table — (PDF) [file pone.0228237.s004.pdf]

S4 Table. Hazard ratios from Cox regressions predicting the risk of divorce by interbirth (IBI) intervals, socioeconomic status (SES), and their interactions.

	Model 1		Model 2	
	HR (95 % CI)	p	HR (95 % CI)	p
1st birth interval				
≤18 months	ref.		ref.	
> 18 months	.72 (.58, .89)	.003	.71 (.52, .96)	.027
2nd birth interval				
≤ 18 months			ref.	
> 18 months			1.15 (.68, 1.93)	.606
SES at age 35				
Farmer	.33 (.16, .65)	.001	.72 (.21, 2.52)	.609
Entrepreneur	.89 (.63, 1.25)	.498	.73 (.28, 1.89)	.515
Upper white collar (ref.)				
Lower white collar	1.01 (.79, 1.29)	.946	.97 (.50, 1.92)	.940
Manual worker	1.19 (.94, 1.52)	.155	1.10 (.56, 2.16)	.778
Student	1.84 (1.27, 2.68)	.001	2.01 (.74, 5.47)	.173
Unknown	2.01 (1.54, 2.63)	<.001	2.21 (1.10, 4.45)	.026
Missing	2.08 (1.51, 2.86)	<.001	2.01 (.89, 4.56)	.094
Interaction between 1st IBI and				
Farmer	1.50 (.73, 3.09)	.270	.93 (.48, 1.81)	.837
Entrepreneur	1.29 (.90, 1.86)	.172	1.34 (.79, 2.27)	.279
Upper white collar (ref.)				
Lower white collar	1.09 (.84, 1.41)	.523	1.29 (.89, 1.89)	.182
Manual worker	.97 (.75, 1.25)	.816	1.14 (.79, 1.64)	.495
Student	.85 (.57, 1.27)	.420	.81 (.47, 1.39)	.443
Unknown	.91 (.69, 1.21)	.525	1.01 (.67, 1.53)	.944
Missing	.91 (.66, 1.26)	.582	.81 (.50, 1.32)	.405
Interaction between 2nd IBI and				
Farmer			.72 (.23, 2.23)	.563
Entrepreneur			1.11 (.47, 2.58)	.816
Upper white collar (ref.)				
Lower white collar			.88 (.48, 1.62)	.685
Manual worker			.96 (.52, 1.76)	.891
Student			1.00 (.40, 2.52)	.999
Unknown			.73 (.38, 1.38)	.326
Missing			1.00 (.47, 2.11)	.998

Note. All models control for birth cohort, marriage length at the start of follow-up, sex, age at first reproduction, and timing of marriage.

Model 1 = Individuals with two children

Model 2 = Individuals with three children, simultaneously controlling for both birth intervals and their interactions with SES.

Supporting Table S4 for Berg V. et al.: Shorter birth intervals between siblings are associated with increased risk of parental divorce; PlosOne 2020
