# Supplementary material for: Shorter birth intervals between siblings are associated with increased risk of parental divorce
Source: PLoS One. 2020 Jan 31;15(1):e0228237. doi: 10.1371/journal.pone.0228237 (PMC6993964; doi:10.1371/journal.pone.0228237)
Supplement: S5 Table — (PDF) [file pone.0228237.s005.pdf]

Table S5. Hazard ratios from Cox regressions predicting the risk of divorce by interbirth (IBI) intervals, age at first reproduction (AFR), and their interactions.

| | Model 1 | | Model 2 | |
|---|---|---|---|---|
| | HR (95% CI) | p | HR (95% CI) | p |
| 1st birth interval | | | | |
| ≤18 months | ref. | | ref. | |
| > 18 months | 0.78 (0.70, 0.87) | .000 | 0.74 (0.64, 0.85) | .000 |
| 2nd birth interval | | | | |
| ≤ 18 months | | | ref. | |
| > 18 months | | | 1.00 (0.82, 1.24) | .965 |
| AFR | | | | |
| < 25 | ref. | | ref. | |
| 25 - 29 | 0.59 (0.51, 0.69) | .000 | 0.47 (0.31, 0.72) | .000 |
| 30 - 34 | 0.49 (0.40, 0.60) | .000 | 0.37 (0.19, 0.72) | .003 |
| ≥ 35 | 0.41 (0.28, 0.61) | .000 | 0.26 (0.03, 2.21) | .217 |
| Interaction between 1st IBI and AFR at | | | | |
| < 25 | ref. | | ref. | |
| 25 - 29 | 0.90 (0.77, 1.05) | .180 | 1.06 (0.84, 1.34) | .606 |
| 30 - 34 | 0.80 (0.65, 1.00) | .049 | 1.64 (1.00, 2.69) | .052 |
| ≥ 35 | 0.87 (0.57, 1.33) | .517 | 0.87 (0.33, 2.24) | .768 |
| Interaction between 2nd IBI and AFR at | | | | |
| < 25 | | | ref. | |
| 25 - 29 | | | 1.14 (0.78, 1.67) | .498 |
| 30 - 34 | | | 0.72 (0.43, 1.23) | .230 |
| ≥ 35 | | | 2.39 (0.32, 18.10) | .398 |

Note. All models control for birth cohort, marriage length at the start of follow-up, sex, and timing of marriage.

Model 1 = Individuals with two children

Model 2 = Individuals with three children, simultaneously controlling for both birth intervals and their interactions with AFR.
