# Supplementary material for: Shorter birth intervals between siblings are associated with increased risk of parental divorce
Source: PLoS One. 2020 Jan 31;15(1):e0228237. doi: 10.1371/journal.pone.0228237 (PMC6993964; doi:10.1371/journal.pone.0228237)
Supplement: S6 Table — (PDF) [file pone.0228237.s006.pdf]

S6 Table. Results from Cox regressions predicting the risk of divorce by birth intervals in all individuals regardless of their final number of children.

	Model 1		Model 2	
	HR (95% CI)	p	HR (95% CI)	p
First interbirth interval				
≤ 18 months	Ref.		Ref.	
18.01 - 24	0.82 (0.76, 0.90)	.000	0.80 (0.70, 0.91)	.001
24.01 - 30	0.74 (0.67, 0.81)	.000	0.79 (0.68, 0.91)	.002
30.01 - 36	0.67 (0.61, 0.75)	.000	0.76 (0.64, 0.90)	.001
36.01 - 42	0.78 (0.70, 0.87)	.000	0.96 (0.79, 1.16)	.654
42.01 - 48	0.77 (0.68, 0.88)	.000	0.89 (0.71, 1.11)	.306
48.01 - 54	0.73 (0.63, 0.84)	.000	1.06 (0.82, 1.37)	.639
54.01 - 60	0.83 (0.71, 0.98)	.024	0.94 (0.69, 1.28)	.687
60.01 - 66	0.83 (0.70, 1.00)	.046	0.85 (0.59, 1.22)	.389
66.01 - 72	0.88 (0.71, 1.09)	.233	0.99 (0.66, 1.49)	.964
> 72	0.88 (0.75, 1.04)	.141	0.91 (0.66, 1.26)	.576
Second interbirth interval				
≤ 18 months			Ref.	
18.01 - 24			1.01 (0.84, 1.21)	.934
24.01 - 30			0.98 (0.81, 1.18)	.797
30.01 - 36			0.85 (0.70, 1.02)	.086
36.01 - 42			0.77 (0.63, 0.94)	.011
42.01 - 48			0.73 (0.59, 0.89)	.002
48.01 - 54			0.77 (0.62, 0.94)	.012
54.01 - 60			0.59 (0.47, 0.74)	.000
60.01 - 66			0.68 (0.55, 0.86)	.001
66.01 - 72			0.61 (0.47, 0.78)	.000
> 72			0.71 (0.60, 0.85)	.000

Note. Both models control for birth cohort, marriage length and quadratic marriage length at the start of follow-up, sex, age at first reproduction, timing of marriage, number of children, and socioeconomic status.

Model 1 = Follow-up starts at the birth of the second child, includes everyone with at least two children.

Model 2 = Follow-up starts at the birth of the third child, includes everyone with at least three children, first two birth intervals simultaneously adjusted.
